# The influence of *Helicobacter pylori* infection on acute coronary syndrome and lipid metabolism in the Chinese ethnicity

**DOI:** 10.3389/fcimb.2024.1437425

**Published:** 2024-09-03

**Authors:** Yizhen Fang, Chunming Fan, Yun Li, Huabin Xie

**Affiliations:** ^1^ Department of Clinical Laboratory, Xiamen Cardiovascular Hospital of Xiamen University, School of Medicine, Xiamen University, Xiamen, China; ^2^ Department of Clinical Laboratory, Xiamen Key Laboratory of Precision Medicine for Cardiovascular Disease, Xiamen, China; ^3^ Blood Transfusion Department, Affiliated Fuzhou First Hospital of Fujian Medical University, Fuzhou, China

**Keywords:** *Helicobacter pylori* infection, acute coronary syndrome, lipid metabolism, Chinese ethnicity, case-control study

## Abstract

**Background:**

Acute coronary syndrome (ACS) patients frequently present a relatively high prevalence of *Helicobacter pylori* (*H. pylori*) infection. *H. pylori* was previously hypothesized to induce ACS through the regulation of lipid levels. However, the risk of *H. pylori*-induced ACS varies significantly among different ethnic groups, and the associations between *H. pylori* and lipid parameters remain unclear. This study aimed to systematically assess the risk of ACS in Chinese populations with *H. pylori* infection while also evaluating the effects of *H. pylori* on lipid parameters.

**Materials and methods:**

A hospital-based case−control study involving 280 participants was conducted. Immunoblotting was used for the detection and genotyping of *H. pylori*. The associations between *H. pylori* and ACS, as well as lipid parameters, were analyzed via the chi-square test and a multiple logistic regression model.

**Results:**

*H. pylori* infection significantly increased the risk of ACS among all participants (adjusted odds ratio (OR) = 4.04, 95% confidence interval (CI): 1.76−9.25, *P* < 0.05), with no associations with virulence factors (*cytotoxin-associated gene A* (*CagA*) or *vacuole toxin geneA* (*VacA*)). Subgroup analysis revealed a significant increase in the risk of ACS among the elderly population aged 56−64 years with *H. pylori* infection. Additionally, a substantial association was observed between *H. pylori* and acute myocardial infarction (AMI). No significant differences were found in lipid parameters, including low-density lipoprotein cholesterol (LDL-C), triglyceride (TG), total cholesterol (TC), high-density lipoprotein cholesterol (HDL-C), and the LDL/HDL ratio, between individuals positive and negative for *H. pylori* infection. Similar results were observed between the ACS group and the control group.

**Conclusions:**

Our study has demonstrated for the first time that *H. pylori* does not significantly impact lipid metabolism but increases the risk of ACS fourfold in the Chinese population (OR = 4.04, 95% CI: 1.76−9.25). Furthermore, the virulence factors of *H. pylori* (*CagA* and *VacA*) may not be involved in the mechanisms by which they promote the development of ACS. This finding provides additional evidence for the association between *H. pylori* and ACS among different ethnic groups and refutes the biological mechanism by which *H. pylori* affects ACS through lipid metabolism regulation. Regular screening for *H. pylori* and eradication treatment in elderly individuals and those at high risk for ACS may be effective measures for reducing the incidence of ACS. Future research should include multicenter randomized controlled trials and explore host genetics and the effects of *H. pylori* on the gut microbiota as potential biological pathways linking *H. pylori* and ACS.

## 1 Introduction

*Helicobacter pylori* (*H. pylori*) has a wide range of adaptation mechanisms that enable its survival in the acidic gastric microenvironment and its colonization of the gastrointestinal tract. According to a recent meta-analysis, the estimated global infection rate of *H. pylori* is 44.3% (Zamani et al., 2018). The highest prevalence of *H. pylori* was found in Africa (79.1%), followed by Latin America, the Caribbean (63.4%), and Asia (54.7%). Conversely, North America (37.1%) and Oceania (24.4%) have the lowest prevalence rates (Hooi et al., 2017). Factors such as dietary habits, economic development, sanitation conditions, and the healthcare environment play crucial roles in influencing the infection rate of *H. pylori*. Therefore, the prevalence of *H. pylori* infection tends to be higher in developing nations than the global average. A meta-analysis of 26 studies involving 103,128 participants in China revealed that the overall estimated prevalence of *H. pylori* infection remained as high as 52.2% from 2000–2016 (Hooi et al., 2017). Despite declines in the general *H. pylori* infection rate in China over the past three to four decades due to interventions, education, improved sanitary conditions, and improved drinking water quality (Li et al., 2020), the public health burden of *H. pylori* infection in China remains substantial, considering that there were still approximately 589 million *H. pylori* infection cases in mainland China in 2022 (Ren et al., 2022). Chinese dietary habits contribute to the transmission of *H. pylori* among family members (Shi et al., 2008; Liu et al., 2023; Li et al., 2024). Additionally, the disparity in regional development has led to a relatively high infection rate of *H. pylori* in rural areas of China (Lu TL. et al., 2022). Since its discovery in 1980 in the stomachs of patients with gastritis, peptic ulcers, and active chronic gastritis, *H. pylori* has been recognized as a significant pathogenic factor contributing to digestive ailments (Machlowska et al., 2020). Recent studies have revealed that the pathogenic role of *H. pylori* extends beyond the gastrointestinal tract, with many extraintestinal diseases showing an underlying correlation with *H. pylori*. For example, a population-based retrospective cohort study demonstrated an association between *H. pylori* infection and an increased risk of Parkinson’s disease (Huang et al., 2018). The involvement of GIT-associated retrograde axonal transport pathways may serve as a crucial mechanism through which *H. pylori* induces neurological diseases (Dobbs et al., 2000; Doulberis et al., 2018). Furthermore, *H. pylori* can colonize the biliary tract and cause chronic active hepatitis. Several cross-sectional studies have reported a high prevalence of *H. pylori* positivity among patients with chronic hepatitis (Ponzetto et al., 2000; Esmat et al., 2012). The mechanism underlying its induction of hepatitis may be associated with the activation of noncanonical nuclear factor-κB (NF-κB), signal transducer and activator of transcription 3 (Stat3), and the mammalian family of mitogen-activated protein kinases (MAPKs) (Pellicano et al., 2008; Cao et al., 2020).

The association between *H. pylori* and the incidence of cardiovascular diseases was first suggested by Mandell et al. in 1994 (Mendall et al., 1994). Since then, numerous researchers have focused on elucidating the mechanisms through which *H. pylori* contributes to atherosclerosis, including immune mediation (Chalubinski et al., 2013) and inflammation involvement (Li et al., 2007). Notably, the presence of *H. pylori* DNA within atherosclerotic plaques provides additional evidence supporting the potential correlation between *H. pylori* and acute cardiovascular events (Ameriso et al., 2001). Over the past decade, the high infection rate of *H. pylori* among patients with acute coronary syndrome (ACS) has attracted the attention of clinical researchers (Azarkar et al., 2011; Khodaii et al., 2011). A case−control study revealed a statistically significant difference in *H. pylori* seropositivity rates between ACS patients and controls (odds ratio (OR) = 3.74, 95% confidence interval (CI): 1.15−12.13) (Miyazaki et al., 2006). Witherell et al. (Witherell et al., 2003) reported that the presence of IgG antibodies against *H. pylori* was more prevalent in patients with acute myocardial infarction (AMI) than in controls (69.4% vs. 56.9%, OR = 1.90) within the American population (Witherell et al., 2003). Additionally, an observational study in Japan demonstrated a significant association between *H. pylori* infection and AMI in younger individuals (OR = 2.97, 95% CI: 1.37−6.41). Notably, most studies did not exclude patients who had previously undergone *H. pylori* eradication treatment, potentially introducing selection bias. The use of enzyme-linked immunosorbent assay (ELISA) kits to detect *H. pylori* increases the risk of false positive results, thus increasing detection bias. A meta-analysis reported a higher rate of *H. pylori* infection in ACS patients than in controls. However, nearly all studies employed ELISA as the detection method for *H. pylori*, with approximately half of the studies rated as low quality (Fang et al., 2019). Furthermore, potential publication bias may lead to the neglect of studies that deny the correlation between *H. pylori* and ACS. Consequently, more rigorous studies are needed to validate the association between *H. pylori* and ACS.

Currently, the association between *H. pylori* and ACS is not well understood. It has been suggested that the microbiome might impact the host’s ability to metabolize lipids, which are essential for absorbing, storing, and generating energy from the diet (Martin et al., 2007). Metabolomic studies have indicated that *H. pylori* infection and eradication can affect various metabolic pathways in the host, including cholesterol metabolism (Ye et al., 2023). Moreover, *H. pylori* infection can disrupt nutritional absorption, potentially leading to abnormal lipid levels (Shiotani et al., 2005). As abnormal lipid levels are associated with an increased risk of ACS (Ference et al., 2017), the regulation of lipid metabolism could be a potential mechanism by which *H. pylori* induces ACS. Some studies have suggested that *H. pylori* infection alters the ratio of low-density lipoprotein cholesterol (LDL-C) to high-density lipoprotein cholesterol (HDL-C) and that *H. pylori* eradication decreases this ratio (Mędrek-Socha et al., 2018). Shimamoto et al. (Shimamoto et al., 2020) reported that *H. pylori* infection elevates LDL-C levels and decreases HDL-C levels. These studies indicate that *H. pylori*-induced alterations in blood lipid profiles might contribute to the development of ACS. However, other studies have refuted the influence of *H. pylori* on blood lipids (Liu et al., 2022). Shiotani et al. (Shiotani et al., 2005) reported no significant differences in triglyceride (TG), total cholesterol (TC), or HDL-C levels between *H. pylori*-infected and uninfected individuals. Furthermore, eradication therapy for *H. pylori* did not significantly impact LDL-C, HDL-C, or TG levels (Upala et al., 2017).

The studies investigating this association involved diverse racial backgrounds, including Asian (Shiotani et al., 2005; Liu et al., 2022; Lu J. et al., 2022), Caucasian (Mędrek-Socha et al., 2018; Ozturk et al., 2024), and Black (Tali et al., 2022) populations. As such, genetic differences may contribute to the variations in study results. However, the heterogeneity of results within the same ethnic groups suggests that genetic background is not the sole reason for these differences. Contradictory conclusions regarding the association between *H. pylori* and LDL-C were drawn from two observational studies conducted in Japan with health check-up populations (Shiotani et al., 2005; Lu J. et al., 2022). Compared with the study by Lu et al. (Lu J. et al., 2022), Shiotani et al. (Shiotani et al., 2005) did not employ rigorous exclusion criteria and failed to exclude patients using lipid-lowering medications or receiving *H. pylori* eradication treatment. This could introduce selection bias and may be a fundamental reason for the contradictory results. Additionally, some studies have utilized less accurate *H. pylori* detection kits, such as ELISA kits (Azarkar et al., 2011; Lim et al., 2019), which reduces the credibility of the results and contributes to heterogeneity among the studies.

The historical tradition of dining in Chinese families typically involves the practice of sharing food from the same plate or bowl or using the same utensils, which could increase the risk of transmitting *H. pylori (* Ding et al., 2022). Consequently, a widespread and sustained epidemic of *H. pylori* infection could pose a significant threat to public health in China. Additionally, owing to notable variations in the risk of ACS caused by *H. pylori* among different racial groups (Fang et al., 2019) and the ongoing debate regarding the association between *H. pylori* and lipid parameters across studies, it remains unclear whether the effect of *H. pylori* on blood lipids is the underlying cause of the increased risk of ACS. Therefore, it is crucial to determine whether *H. pylori* infection increases the risk of ACS in the Chinese population, with the aim of reducing the incidence of ACS. Our study sought to systematically evaluate the risk of ACS in Chinese populations with *H. pylori* infection and assess the impact of *H. pylori* on lipid parameters. By doing so, we anticipate offering additional evidence for the association between *H. pylori* and ACS across different ethnic groups, as well as providing recommendations for lipid management in *H. pylori* carriers. Consequently, this research will guide clinicians in selecting appropriate populations for *H. pylori* screening and eradication, thus contributing to the prevention of ACS and the refinement of treatment guidelines for *H. pylori*.

## 2 Materials and methods

### 2.1 Ethics statement

The case−control study design carries inherent risks of selection bias and challenges in controlling for confounding factors. Nevertheless, this design is well suited for studying diseases with long latency periods and simultaneously investigating the associations between multiple exposure factors and diseases. Hence, we employed a hospital-based case−control study design that adhered to the guidelines of the STROBE statement (von Elm et al., 2007). The Ethics Committee of Xiamen Cardiovascular Hospital of Xiamen University granted approval for this project, which was conducted in accordance with the principles outlined in the Declaration of Helsinki. Prior to their participation in the study, prospective participants were fully informed about the research details by the principal investigator and provided with the opportunity to freely choose whether to take part in the experiment. Finally, informed consent forms were signed by all participants.

### 2.2 Study subjects

Case group: Patients who visited Xiamen Cardiovascular Hospital of Xiamen University between January 2020 and April 2022, where they were diagnosed with ACS. The inclusion criteria were as follows: (1) Patients with initial diagnoses of ACS, including unstable angina (UA), myocardial infarction without ST elevation (NSTEMI), and myocardial infarction with ST segment elevation (STEMI). The diagnoses of ACS were made in accordance with the current guidelines (Collet et al., 2021; Ibanez et al., 2018). (2) Age ≥18 years. The exclusion criteria were as follows: (1) Patients with tumors. (2) Patients with autoimmune diseases. (3) Patients who had undergone recent surgery. (4) Patients who had previously received *H. pylori* eradication therapy. (5) Patients who had previously taken lipid-lowering drugs. (6) Patients with a family history of hyperlipidemia.

Control group: The population underwent health examinations at Xiamen Cardiovascular Hospital of Xiamen University between January 2020 and April 2022. The inclusion criteria were as follows: (1) Apparently normal healthy population undergoing physical examination. (2) Age ≥18 years. The exclusion criteria were as follows: (1) Received *H. pylori* eradication therapy. (2) Patients with tumors. (3) Recent surgical history. (4) Patients who previously took lipid-lowering drugs. (5) History of autoimmune diseases. (6) History of cardiovascular diseases. (7) Patients with a family history of hyperlipidemia.

The inclusion criteria for both the control group and the case group included adults without any restrictions on region, ethnicity, sex, culture, or economic conditions. The case group comprised newly diagnosed ACS patients, which helped to reduce prevalence-incidence bias. The use of internationally recognized standards for diagnosing ACS helped minimize diagnostic bias. Additionally, both groups implemented strict and uniform exclusion criteria to eliminate the same types of influencing factors. Researchers had made every effort to persuade eligible individuals to participate in the study to reduce nonresponse bias. These methods effectively minimized potential selection bias, ensuring that the control group and case group were somewhat representative, thereby enhancing the external validity of the study.

Although completely eliminating residual confounding in observational studies is difficult, we used stratified analysis to minimize its impact as much as possible.

### 2.3 Demographic characteristics and sample size

The demographic characteristics of the subjects, including age, sex, history of hypertension, smoking history, and history of diabetes, were collected for the purpose of multivariate and stratified analyses to reduce selection bias. Sample size estimation and power analysis were performed via PASS software (version 15.0.05), with a power calculation (1-β) of 0.9 and an alpha (α) level of 0.05. According to a meta-analysis (Hooi et al., 2017; Fang et al., 2019), the overall infection rate of *H. pylori* in the Chinese ethnicity group was 52.2%, with an OR for *H. pylori* infection in ACS patients of 2.45. The alternative hypothesis was two-sided.

### 2.4 Blood sample collection

Following the subjects’ consent, all participants fasted for 12 hours, after which blood samples were collected the next day between 9 and 10 AM. A volume of 5 ml of fasting venous blood was obtained from the antecubital fossa of the subjects via serum separation tubes. The collected blood samples were left to stand at room temperature for 30 minutes and then centrifuged at 4000 rpm for 10 minutes to separate the serum from the formed elements. The extracted serum was stored in tubes and frozen at -80°C for subsequent experimental analysis.

### 2.5 Laboratory analysis

A Beckman AU5800 analyzer was utilized for the detection of LDL-C, HDL-C, TG, and TC. The enzymatic photometric method was employed for TC detection, whereas the homogeneous method was used for the detection of LDL-C and HDL-C. The TG concentration was determined via the GPO-POD method. Prior to sample testing, internal quality control (IQC) was performed, and the IQC results were required to adhere strictly to Westgard rules. All samples within the same batch were tested within one hour.

The confirmation of dyslipidemia was based on increases in TC (≥6.20 mmol/L), LDL-C (≥4.13 mmol/L), and TG levels (≥2.25 mmol/L) or a decrease in HDL-C (≤1.03 mmol/L) (Kopin and Lowenstein, 2017). This standard was established on the basis of findings from long-term cohort studies conducted within the Chinese population (Joint Committee on the Chinese Guidelines for Lipid Management, 2023).

### 2.6 Determination of *H. pylori*

Immunoblotting, a rapid and accurate noninvasive sensitive serologic test, was employed to detect antibodies against specific *H. pylori* antigens, including urease A and B subunits (UreA and UreB), *cytotoxin-associated gene A* (*CagA*), and *vacuolating cytotoxin A* (*VacA*). Compared with those of the ^13^C urea breath test (UBT), the sensitivity, specificity, positive predictive value, negative predictive value, and concordance rate of immunoblotting for *H. pylori* detection are 97.7%, 86.8%, 94.0%, 94.6%, and 94.2%, respectively (Dong et al., 2016). The participants’ *H. pylori* infection status was determined by immunoblotting (the *H. pylori* typing kit for antibody detection was produced by Shenzhen Boluote Biological Products Co., Ltd.).

*H. pylori* strains have been classified into Type I and Type II strains on the basis of the presence of *VacA* toxin and/or *CagA* protein. In accordance with the antibody typing results for *H. pylori*, *H. pylori* infection can be categorized as *H. pylori*-I infection and *H. pylori*-II infection. The diagnosis of *H. pylori*-II infection required positive UreA or/and UreB results. On the other hand, positive *CagA*, *VacA*, or both antibodies indicated *H. pylori*-I infection. If all four antibodies yielded negative results, it was deemed a non-*H. pylori* infection. *H. pylori*-I can produce cytotoxins (*Caga* and *Vaca*), which are associated with gastric diseases such as gastric and duodenal ulcers, lymphoma, and gastric cancer. *H. pylori*-I is typically more virulent and infectious. Conversely, *H. pylori*-II does not produce cytotoxins, and most infected patients develop chronic superficial gastritis, which is generally less virulent, less infectious, and causes less damage to the stomach.

### 2.7 Missing data

In cases where clinical characteristics were missing, the corresponding author contacted participants to obtain the necessary information. For missing serum detection data, the corresponding author contacted participants to perform resampling. If participants could not be reached, cases with missing data were excluded from the analysis.

### 2.8 Statistical analysis

Statistical analysis was performed via SPSS 20.0 software. Continuous variables were presented as the means ± standard deviations, whereas categorical variables were expressed as percentages. Comparisons of normally distributed continuous variables were conducted via analysis of variance (ANOVA), whereas the Mann–Whitney U test was used for skewed distributed continuous variables. The chi-square test or Fisher’s exact probability method was employed to compare categorical variable data. Logistic regression analysis was used for multivariate analysis to estimate the adjusted OR and 95% CI. *Post hocp* values were adjusted for multiple comparisons via Bonferroni correction. A significance level of less than 0.01 was used to identify statistical significance.

Baseline variables considered clinically relevant or showing a univariate association with the outcome were entered into logistic regression models. Initially, a crude model (Model 1) was established, which was unadjusted and included only the exposure factor (*H. pylori*) and the outcome variable, without any covariates. A partially adjusted model was subsequently developed, built on Model 1, by incorporating potential confounding factors that may affect the outcome, such as demographic characteristics, comorbidities, and laboratory test results, for multivariate analysis. Depending on the covariates included, multiple partially adjusted models were established:

Model 2: adjustment for age, sex, and smoking status based on Model 1.

Model 3: incorporation of variables such as hypertension and diabetes into Model 2.

Finally, a fully adjusted model (Model 4) was created, further adjusting for the concentrations of TC, TG, LDL-C, and HDL-C based on Model 3, to include all potential confounding factors that may influence the association between *H. pylori* and the outcome variable to comprehensively estimate the OR.

## 3 Results

### 3.1 Characteristics of the study participants

The sample size calculation results indicated that both the control group and the case group should have a minimum of 111 cases. A total of 280 subjects were included in this study, comprising 140 ACS patients and 140 healthy controls, with a power analysis result of 0.95373. Healthy controls were selected from individuals without cardiovascular diseases, tumors, or autoimmune diseases and could represent the normal healthy control population for cardiovascular-related research in China. The clinical information was summarized in **Table 1**. The case group consisted of 111 patients with STEMI, 22 patients with NSTEMI, and 7 patients with UA. The average age of the patients in the case group was 59.20 ± 12.98 years, with 35.71% of individuals aged 65 years or older. The average age of the control group was 50.96 ± 12.49 years, with 7.86% of individuals aged 65 years or older. The male proportion in the case group was 81.43%, whereas in the control group, it was 47.86%. In the case group, the proportions of males, individuals with hypertension, individuals with diabetes, and smokers, as well as the average age and mean concentrations of serum TG, TC, and LDL-C, were significantly greater than those in the control group. However, the serum HDL-C concentration was significantly lower. Hypertension, smoking, and diabetes are well-established traditional risk factors for cardiovascular disease. Therefore, baseline indicators for these factors were significantly greater in the case group than in the control group. Furthermore, there was a significant sex difference between the two groups, which could be attributed to variations in cardiovascular risk factors, such as smoking behaviors, between sexes, resulting in a greater incidence of ACS among men than among women. Gender imbalance may lead to research findings that disproportionately represent the male population. Therefore, it was crucial to address the influence of gender bias through regression analysis models.

**Table 1:** Clinical characteristics by group.

	ACS group	Control group	*P* value
Age years	59.20 ± 12.98	50.96 ± 12.49	<0.05
Man (%)	81.43%	47.86%	<0.05
Hypertension %	53.57%	10%	<0.05
Current smoking %	66.43%	23.57%	<0.05
diabetes %	18.57%	5.71%	<0.05
LDL Cholesterol mmol/L	3.85 ± 0.86	3.35 ± 0.78	<0.05
HDL Cholesterol mmol/L	1.15 ± 0.33	1.32 ± 0.30	<0.05
Total Cholesterol mmol/L	5.55 ± 1.22	5.17 ± 1.01	<0.05
Triglyceride mmol/L	2.11 ± 1.86	1.50 ± 1.31	<0.05
Hp (positive/negative)	113/27	90/50	<0.05
Caga (positive/negative)	84/29	75/15	>0.05
Vaca (positive/negative)	63/50	55/35	>0.05

### 3.2 Association between *H. pylori* infection and ACS

The prevalence of *H. pylori* was greater in the case group (80.71%) than in the control group (64.29%), with a significant difference (OR = 2.32, 95% CI: 1.35−4.00). *H. pylori* infection often leads to changes in the body’s internal environment, such as an increase in inflammatory factors (Chen et al., 2013), oxidative stress (Elizalde et al., 1997), and endothelial dysfunction (Benjamin et al., 2004). When carriers of *H. pylori* are continuously exposed to these factors, it can easily result in atherosclerosis, subsequently triggering ACS. Therefore, it is not uncommon to observe a higher infection rate of *H. pylori* among ACS patients. *H. pylori* was significantly associated with ACS in all the models after adjusting for different risk factors for ACS (**Table 2**). According to the fully adjusted model (Model 4), *H. pylori* infection significantly increased the risk of ACS (OR = 4.04, 95% CI: 1.77−9.25). This result is consistent with many previous reports (Witherell et al., 2003; Miyazaki et al., 2006) and indicates that *H. pylori* may be an independent risk factor for ACS. The contribution rates of sex, age, hypertension, diabetes, smoking, TC, TG, HDL, LDL and *H. pylori* in Model 4 were 0.50%, 5.08%, 17.73%, 6.23%, 9.94%, 13.87%, 2.69%, 5.81%, 26.81%, and 11.34%, respectively. The wider CI in Model 4 indicates variability in the results, which may arise from genetic differences among individuals. Increasing the sample size from different sources would help reduce this variability, thereby enhancing the reliability of the results.

**Table 2:** Multivariate logistic regression analysis for the effect of *H. pylori* on ACS after adjusting multiple confounding factors.

Multivariate logistic regression analysis	S.E	OR	95%CI	*P* value
Model 1	0.28	2.32	1.35-4.00	<0.05
Model 2	0.32	2.51	1.34-4.69	<0.05
Model 3	0.35	2.51	1.25-5.02	<0.05
Model 4	0.42	4.04	1.77-9.25	<0.05

Model 1: Only included the status of *H. pylori* infection. Model 2: Adjustment for age, gender and smoking based on Model 1. Model 3: Incorporated variables such as hypertension and diabetes into Model 2. Model 4: Further adjustment for the concentrations of TC, TG, LDL-C and HDL-C based on Model 3.

Although the use of multivariate models to correct for confounders makes the results more reliable, there are still some residual confusions that could affect our results. For example, we included smoking as a confounding factor, but the duration of smoking and daily cigarette consumption could have different effects on the results. Additionally, stress is an important factor affecting cardiovascular disease. However, it is difficult to quantify the stress variable, so we cannot avoid its impact on the results. Therefore, it is important to maintain a cautious attitude toward the results.

The frequency of antibodies against *CagA* and *VacA* antigens did not significantly differ between the case group and the control group (*CagA*: 74.34% vs 83.33%, *VacA*: 55.75% vs 61.11%). Shimoda et al. (Shimoda et al., 2016) first reported the detection of *CagA* in serum-derived exosomes from patients infected with *CagA*-positive *H. pylori*, suggesting a pathway through which the virulence factor *H. pylori* influences extraintestinal diseases. However, a recent study revealed no difference in the levels of *CagA* in the circulation between *H. pylori*-infected subjects and control subjects (Imoto et al., 2022). Thus, there remains considerable controversy regarding whether the virulence factors of *H. pylori* can influence extraintestinal diseases through extraintestinal pathways. Our research results indicate that the two virulence factors of *H. pylori* (*CagA* and *VacA*) are not the main factors affecting ACS. The mechanism by which *H. pylori* promotes the occurrence of ACS may not be limited to specific strains but may stem from the common biological characteristics of all *H. pylori* strains in the Chinese population.

The subgroup analysis of ACS patients revealed a significant association between *H. pylori* infection and an increased risk of STEMI and NSTEMI in the multivariate analysis (**Table 3**). The power analysis results for the STEMI, NSTEMI, and UA subgroups were 0.93166, 0.50923, and 0.22073, respectively, suggesting that the results for the STEMI subgroup were more reliable. AMI and UA are the two subtypes of ACS. Research had shown that when the infection rates of *H. pylori* were not significantly different between AMI patients and those with UA, the antibody titers of *H. pylori* and C-reactive protein (CRP) levels in AMI patients were significantly greater than those in UA patients (Ozdogru et al., 2007). This may suggest that *H. pylori* infection could have a greater impact on the development of AMI. Our results also support a positive correlation between AMI and *H. pylori*, especially in STEMI patients. The results adjusted for confounding factors indicate that the risk of developing STEMI is 4.58 times greater in individuals with *H. pylori* infection than in those without infection. Age stratification analysis revealed a significantly greater prevalence of *H. pylori* infection among ACS patients aged 56–64 years than in the control group (**Table 4**). *H. pylori* is less likely to cause ACS in younger age groups, possibly because of a stronger autoimmune response in these individuals. However, the power analysis results for the Quartile 1 to Quartile 4 subgroups were unsatisfactory, each being less than 0.5. This may be due to the insufficient sample sizes across different age groups, leading to a decrease in the reliability of the results.

**Table 3:** The impact of *H. pylori* on various types of ACS.

Subgroups of patients	Univariate analysis			Multivariate analysis^†^		
	OR	95%CI	*P* value	OR	95%CI	*P* value
ACS	2.32	1.35-4.00	<0.05	4.04	1.77-9.25	<0.05
STEMI	2.69	1.47-4.92	<0.05	4.58	1.84-11.38	<0.05
NSTEMI	1.89	0.66-5.43	0.33	6.31	1.05-37.80	<0.05
UA	0.74	0.16-3.44	0.70	5.47	0.06-467.59	0.454

^†^Adjustment for age, gender, hypertension, smoking, diabetes, the concentrations of TC, TG, LDL-C and HDL-C.

**Table 4:** *H. pylori* positive rate by age group.

	ACS group	Control group	OR (95% CI)	*P* value
Quartile 1 (≤47y)				
Hp positive	17	34	0.67 (0.23-1.89)	0.59
Hp negative	9	12		
Quartile 2 (48-56y)				
Hp positive	34	27	3.46 (0.99-12.10)	0.08
Hp negative	4	11		
Quartile 3 (57-64y)				
Hp positive	23	21	8.76 (2.30-33.40)	<0.05
Hp negative	3	24		
Quartile 4 (≥65y)				
Hp positive	39	8	1.33 (0.30-5.88)	0.70
Hp negative	11	3		

### 3.3 Lipid parameters among all participants

The results of the quality control for lipid profile testing adhered to the Westgard rules, with the IQC results falling within the target range of ±2 standard deviations (SD) for that particular day. As shown in **Table 5**, the lipid parameters were classified as either *H. pylori*-positive or *H. pylori*-negative. No significant differences were found in the levels of TG, TC, LDL-C, or HDL-C or the LDL/HDL ratio between individuals who tested positive or negative for *H. pylori*. Additionally, compared with *H. pylori*-negative ACS patients, *H. Pylori*-positive ACS patients did not present significant increases in TG, TC, LDL-C, HDL-C, or the LDL/HDL ratio. Similar results were observed in the control group. However, although **Table 5** categorized the groups on the basis of health status to mitigate the impact of confounding factors to some extent, it did not account for variables such as age, sex, and smoking, which may lead to biased results.

**Table 5:** Correlation between *H. pylori* infection and lipid parameters.

	All participants			ACS patients			Control individuals		
	*H. pylori* positive	*H. pylori* negative	*P* value	*H. pylori* positive	*H. pylori* negative	*P* value	*H. pylori* positive	*H. pylori* negative	*P* value
TC	5.36 ± 1.15	5.35 ± 1.11	0.77	5.55 ± 1.26	5.53 ± 1.08	0.84	5.13 ± 0.95	5.26 ± 1.12	0.44
HDL-C	1.22 ± 0.34	1.27 ± 0.29	0.21	1.15 ± 0.35	1.14 ± 0.24	0.98	1.31 ± 0.31	1.35 ± 0.30	0.66
LDL-C	3.58 ± 0.85	3.63 ± 0.88	0.50	3.81 ± 0.87	3.97 ± 0.83	0.26	3.30 ± 0.73	3.45 ± 0.85	0.30
TG	1.90 ± 1.80	1.56 ± 1.07	0.21	2.14 ± 1.96	1.98 ± 1.37	0.91	1.59 ± 1.53	1.33 ± 0.79	0.60
LDL/HDL	3.11 ± 1.0	3.01 ± 1.05	0.23	3.49 ± 0.92	3.65 ± 1.18	0.64	2.63 ± 0.80	2.66 ± 0.79	0.91

Moreover, the subgroup analysis by sex revealed that *H. pylori* infection did not result in significant changes in lipid levels in either males or females. Logistic regression analysis was used to adjust for variables such as age, smoking status, and sex (**Table 6**). The impact of sex differences on lipid metabolism is well recognized (Link and Reue, 2017). Mumford et al. (Mumford et al., 2010) reported that estrogen was associated with decreased levels of TC and LDL-C, as well as increased levels of HDL-C. Animal models had shown that the dosage of the X chromosome affected HDL-C levels (Zore et al., 2018). Therefore, the analysis of different sex subgroups can provide a more objective reflection of the true associations between *H. pylori* and blood lipids. Consequently, we assert that *H. pylori* may not have the ability to alter lipid metabolism, leading to consistent results regardless of sex.

**Table 6:** Multivariate logistic regression analysis to predict the effect of *H. pylori* infection on lipid parameters.

Dyslipidemia	Univariate analysis			Multivariate analysis^†^		
	OR	95%CI	*P* value	OR	95%CI	*P* value
TC (≥6.20 mmol/L)	1.40	0.71-2.77	0.33	1.37	0.69-2.72	0.37
TG (≥2.25 mmol/L)	1.45	0.74-3.02	0.26	1.45	0.71-2.98	0.31
HDL (≤1.03mmol/L)	1.14	0.63-2.06	0.66	1.01	0.53-1.91	0.99
LDL (≥4.13 mmol/L)	0.98	0.52-1.84	0.94	0.94	0.50-1.79	0.86
LDL/HDL (≥4.01)	1.05	0.51-2.16	0.89	0.95	0.45-2.02	0.90

^†^Adjustment for age, gender and smoking.

Dyslipidemia is a condition that results from a combination of various factors, including age, sex, smoking status, comorbidities, and genetics. Some research provided evidence linking *H. pylori* infection to dyslipidemia, regardless of potential confounders (Eslami et al., 2017; Seo et al., 2020). A prospective study conducted by Elizalde et al. (Elizalde et al., 2002), which rigorously controlled for confounding factors, yielded results consistent with our findings. Furthermore, the persistent inflammatory response caused by *H. pylori* colonization is considered an important mechanism that leads to changes in individual lipid levels. However, studies conducted in different populations to validate this hypothesis had produced conflicting results (Danesh and Peto, 1998; Strachan et al., 1998; Hoffmeister et al., 2001). Hence, it is important to analyze the association between *H. pylori* and dyslipidemia in the context of specific ethnic backgrounds to avoid inaccurate conclusions resulting from excessive confounding factors.

Many studies involving diverse ethnic groups often yield disparate results (Park et al., 2005; Iwai et al., 2019; Hashim et al., 2022), suggesting potential ethnic variations in the regulatory effects of *H. pylori* on blood lipids. Our study focused predominantly on individuals from the Chinese population, which could lead to inconsistencies compared with prior studies conducted in non-Chinese populations. Some studies utilizing ELISA test kits may encounter substantial discrepancies in detection results due to variations in the antibodies employed by different manufacturers. These discrepancies may serve as the primary cause of the disparities between our research findings and those of other studies. Additionally, certain studies employed less rigorous exclusion criteria than ours did, potentially introducing excessive confounding factors and consequently leading to misleading conclusions (Iwai et al., 2019; Hashim et al., 2022).

## 4 Discussion

*H. pylori* can be transmitted among individuals through direct contact with saliva, vomiting, or feces. The increased prevalence of *H. pylori* infection is often associated with factors such as overcrowded living conditions, contaminated drinking water, and communal dining practices. Consequently, the infection rate of *H. pylori* varies across regions and countries, ranging from approximately 30%-40% in developed countries to as high as 80% in developing countries (Atherton, 2006). Our study revealed an infection rate of 64.29% *H. pylori* in the general population of China, which closely aligns with prior reports on the infection rate of *H. pylori* in the Chinese population (Bai et al., 2023).

In addition to being associated with well-known gastrointestinal diseases and gastric cancer, *H. pylori* has been found to be closely associated with various extraintestinal diseases, including neurological disorders, autoimmune diseases, and cardiovascular diseases. ACS, a severe and common cardiovascular disease, has become a significant cause of mortality in China. Controlling and avoiding independent risk factors for ACS is crucial for preventing and reducing its incidence and mortality. In recent years, *H. pylori* has been recognized as a potentially significant independent risk factor for ACS (Wang et al., 2020; Wang et al., 2023). Although most evidence supports a positive correlation between *H. pylori* and ACS, the relevance of *H. pylori* to ACS varies among different ethnic groups and geographical regions (Fang et al., 2019).

Our research revealed that the infection rate of *H. pylori* in patients with ACS was significantly higher than that in the normal population (80.71% vs 64.29%). After adjusting for potential confounding factors, the risk of ACS significantly increased with *H. pylori* infection (adjusted OR = 4.04, 95% CI: 1.77–9.25). These findings are consistent with studies conducted in multiple regions, including the Middle East (Rahmani et al., 2017) and Europe (Aceti et al., 2004). However, studies involving Asian populations often do not align with our findings because of the use of less accurate methods for detecting *H. pylori* and the lack of stringent exclusion criteria. Additionally, these studies often do not adjust for confounding factors, which compromised the reliability of the results (Chaudhury et al., 2004; Ikeda et al., 2013).

Importantly, although we used a multivariable model, there may still be unaccounted residual confounding factors, such as lifestyle, dietary preferences, and stress. Although these confounding factors are difficult to quantify, they may still have potential effects on the results. Moreover, the results of a single-center study may not necessarily be representative of the entire population. Therefore, although we found a positive correlation between *H. pylori* and ACS, this conclusion applies only to the Chinese population.

Certain clinical studies had reported a significantly higher infection rate of *H. pylori* and serum positivity rate of *CagA* antibodies in patients with ACS than in the normal population (Khodaii et al., 2011). Gunn et al. (Gunn et al., 2000) reported that the association between chronic *H. pylori* infection and the risk of AMI was limited to *CagA*-positive strains. However, a recent study (Wärme et al., 2023) revealed no significant difference in *CagA* antibody levels between AMI patients and the normal population. Various studies had demonstrated the variability of the role of *H. pyloriCagA* among different populations (El Khadir et al., 2021). Since our study focused on a different population than those in previous studies did, we deduced that the variability in the risk of ACS associated with the *CagA* virulence factor may be due to genetic background differences. Furthermore, most studies had indicated that the effects of the *CagA* and *VacA* virulence factors were confined primarily to the gastrointestinal tract (Ferreira et al., 2014; Nejati et al., 2018). Therefore, we hypothesized that these factors may play limited pathogenic roles in extraintestinal diseases. Our study revealed that the *CagA* and *VacA* virulence factors were not the main contributors to the increased risk of ACS caused by *H. pylori* infection in the Chinese population. This finding suggests that the mechanism by which *H. pylori* induces ACS may be based on the shared biological characteristics of all strains rather than specific factors of certain strains.

Through subgroup analysis, our study demonstrated a significant correlation between *H. pylori* and AMI, whereas no significant correlation was found with UA. We believe that an adequate number of patients with AMI accurately reflects the association between AMI and *H. pylori*, which aligns with the findings of previous studies conducted on non-Chinese populations (Pellicano et al., 2002; Khodaii et al., 2011; Nakić et al., 2011). However, the limited sample size of UA patients may introduce statistical errors, potentially obscuring the true association between UA and *H. pylori*. *H. pylori* infection causes metabolic changes with age (Shan et al., 2017; Mut Surmeli et al., 2019). The age-stratified analysis in our study revealed a significant increase in the risk of ACS associated with *H. pylori* in the 56–64 years age group, indicating that *H. pylori* is more likely to increase the risk of ACS in the geriatric population. However, similar results were not observed in the 65 years and above age group. We speculate that the limited representation of individuals aged 65 years and above in the control group, constituting only 7.86% of the total control group, may lead to deviations from the true values.

Local and systemic inflammation triggered by microorganisms and infectious pathogens is a recognized risk factor for cardiovascular diseases (Franceschi et al., 2014). *H. pylori* infection often results in a persistent chronic inflammatory state, which could contribute to the progression of atherosclerosis (Kawano et al., 2001; Babes et al., 2021). This chronic inflammatory process may indirectly cause endothelial cell injury, thereby promoting the development of atherosclerosis (Chmiela et al., 2015; Krupa et al., 2021). The lipopolysaccharide of *H. pylori* can stimulate the production of various inflammation-related cytokines associated with ACS, such as tumor necrosis factor-α (TNF-α), interleukin-1 (IL-1), and interleukin-6 (IL-6) (Manolakis et al., 2007). These *H. pylori*-activated cytokines can directly or indirectly facilitate the inflammatory process in the arterial wall (Pieniazek et al., 1999; Tamer et al., 2009). According to the immune hypothesis, *H. pylori* infection may trigger an autoimmune response in the host through antigenic mimicry, ultimately aggravating vascular endothelial injury (Rechciński et al., 2002). The high sequence homology between *H. pylori* heat shock proteins (HSPs) and human HSPs can lead to an abnormal autoimmune reaction, consequently promoting the progression of atherosclerosis (Benjamin and McMillan, 1998). Furthermore, recent studies suggest that dysregulation of lipid metabolism may be a primary mechanism by which *H. pylori* induces ACS. *H. pylori* infection may influence lipid metabolism by increasing the levels of inflammatory cytokines (Feingold and Grunfeld, 1992; Georges et al., 2003). Elevated levels of TNF-α caused by *H. pylori* infection can inhibit lipoprotein lipase activity (Makoveichuk et al., 2017), resulting in the transfer of lipids from tissues and increased levels of TG in the serum, along with decreased levels of HDL-C (Sheu et al., 2000; Kucukazman et al., 2009). Multiple studies have indicated that *H. pylori* infection-induced gastric mucosal atrophy disrupts the balance between leptin and ghrelin in the body, potentially leading to imbalances in nutritional absorption and abnormal changes in blood lipids (Azuma et al., 2001; Osawa et al., 2005; Shiotani et al., 2005; Roper et al., 2008). Additionally, *H. pylori* infection can increase fatty acid synthesis in the liver and affect lipolysis (Papamichael et al., 2009).

Although the regulation of lipid metabolism was previously considered a potential mechanism through which *H. pylori* induces ACS, our findings do not support this hypothesis. In the Chinese population, we found that *H. pylori* infection did not significantly affect lipid parameters. This phenomenon was observed not only in healthy individuals but also in ACS patients. Furthermore, we observed no significant correlation between *H. pylori* and lipid parameters in different sex groups. Sotuneh et al. (Sotuneh et al., 2014) noted that the high prevalence of abnormal lipid metabolism and *H. pylori* infection in the population could lead to a spurious correlation between these phenomena. Additionally, we believe that the ability of *H. pylori* to regulate lipid metabolism by influencing nutrient absorption in the digestive system may have been overestimated. Several studies have shown no notable change in body weight following the eradication of *H. pylori* (Kawano et al., 2001; Jang et al., 2008).

Currently, the majority of studies confirming the association between *H. pylori* and lipid metabolism are observational studies involving individuals undergoing physical examinations (Sun et al., 2016; Eslami et al., 2017; Lu et al., 2018; Kwon et al., 2022). However, these studies have deficiencies in their exclusion criteria, as they do not adequately consider factors that influence blood lipid levels, such as the use of lipid-lowering medications and a family history of hyperlipidemia. This lack of consideration may introduce selection bias. Additionally, some studies utilize rapid urease tests and ELISA, which have lower detection accuracy for *H. pylori* diagnosis, leading to measurement bias (Eslami et al., 2017; Lim et al., 2019). Confounding factors are also not fully considered in some studies, causing confounding bias (Sun et al., 2016; Lu et al., 2018; Kwon et al., 2022). These aforementioned defects in previous studies may explain the inconsistency with our research results. Sotuneh (Sotuneh et al., 2014) et al. and Naja (Naja et al., 2012) et al. suggested that the type of *H. pylori* strain and host genetic background may render *H. pylori* unable to play a role in lipid metabolism. Furthermore, lipid metabolism is closely associated with gene polymorphisms (Palacio Rojas et al., 2017). Therefore, genetic differences among different study populations may contribute to the discrepancies between our results and those of previous studies. The diagnostic criteria for dyslipidemia are constantly being updated, meaning that conclusions drawn using the latest diagnostic standards may differ from results obtained using old standards.

To date, several studies involving diverse regions and ethnic groups have produced results that are consistent with our findings. In a cross-sectional study of the elderly population, no significant correlation was found between *H. pylori* infection and blood lipid levels (Sotuneh et al., 2014). A study conducted in an African population further confirmed the absence of a correlation between *H. pylori* and TC, TG, and LDL-C (Longo-Mbenza et al., 2012). Ozturk (Ozturk et al., 2024) et al. also reported that there was no significant correlation between *H. pylori* infection and either traditional or nontraditional novel lipid indicators. Furthermore, no significant changes in blood lipid levels were observed in individuals infected with *H. pylori* following eradication therapy (Elizalde et al., 2002). Thus far, most of the populations examined with negative results have been Caucasians and Africans, and no relevant reports have been published regarding the Chinese population. Therefore, our study is the first to refute the impact of *H. pylori* on lipid metabolism in the Chinese population, further supplementing the evidence that there is no correlation between *H. pylori* and lipid parameters across different ethnicities.

Case−control studies, as a type of observational research, have inherent limitations. For example, they cannot randomly assign study subjects, effectively address confounding and bias issues, or clearly define the temporal sequence between exposure factors and disease occurrence. Therefore, causal associations cannot be directly inferred from observational studies. However, our study addressed various confounding factors and employed subgroup analysis and regression analysis to control for bias. Additionally, the formation of antibodies following *H. pylori* infection requires a certain amount of time. Consequently, the selected detection method ensured that *H. pylori* infection occurred prior to the onset of ACS, establishing a foundation for inferring a causal association between the two. Therefore, the measures implemented in this study effectively address the inherent limitations of observational research.

Pathogenic microorganisms impose a significant burden on cardiovascular diseases (Babeş et al., 2021). Considering the high prevalence of *H. pylori* carriers in China, it is crucial to pay more attention to the association between *H. pylori* and cardiovascular diseases. This study is the first to establish a positive correlation between *H. pylori* and ACS in the Chinese population, and debunks the hypothesis that *H. pylori* regulates lipid metabolism. Currently, the prevention of ACS primarily focuses on controlling traditional risk factors, overlooking the impact of pathogen burden on cardiovascular disease. Therefore, we recommend recognizing *H. pylori* as a risk factor for ACS. Elderly individuals and those at high risk for ACS should undergo regular screening for *H. pylori* and receive eradication therapy. On the other hand, individuals with low cardiovascular risk who are infected with *H. pylori* may not require strict lipid management. Nonetheless, actively addressing *H. pylori* infection remains essential.

Several limitations were considered in this study. First, an epidemiological survey revealed that the prevalence of *H. pylori* infection among elderly individuals in China is less than 40% (Zhou and Lyu, 2023). The control group had an insufficient number of elderly participants (aged ≥65 years), resulting in inaccurate and high infection rates (72.73%), which may have obscured the positive correlation between *H. pylori* and ACS. Second, there were fewer patients with UA in the ACS group, so the subgroup analysis results may not accurately reflect the association between *H. pylori* and UA. Third, the impact of genetic background among diverse populations is a crucial factor that requires careful consideration. Therefore, our results could only be cautiously applied to the Chinese population. Fourth, despite considering various confounding factors, our approach was not sufficiently comprehensive. Some confounding factors, such as dietary habits, lifestyle choices, and psychological stress, whereas challenging to quantify, may still have an impact on our results. Fifth, there were limited matching variables between the control group and the case group. Although we used regression analysis and subgroup analysis to minimize the influence of confounding factors, potential biases still exist. Finally, a multiple-center study revealed that the sensitivity and specificity of immunoblotting for diagnosing *H. pylori* infection were 97.7% and 86.8%, respectively, with a concordance rate of 94.2% compared with UBT (Dong et al., 2016). However, serum antibody levels can remain elevated for months to years after the eradication of *H. pylori* infection, which may introduce bias in the correlation between *H. pylori* and ACS. Although the immunoblotting method had good accuracy and our study excluded individuals who had undergone *H. pylori* eradication therapy, from a rigorous methodological perspective, it should be noted that the inferences of this study may be limited to exposure to *H. pylori* infection rather than active infection.

Future multicenter randomized controlled trials should be conducted to further investigate whether *H. pylori* is an independent risk factor for ACS. Exploring host genetics and the effects of *H. pylori* on the gut microbiota are interesting directions for future research into the biological pathways associated with *H. pylori* and ACS.

## 5 Conclusion

In conclusion, our study has demonstrated, for the first time, that *H. pylori* does not significantly impact lipid metabolism. However, it increases the risk of ACS by fourfold in the Chinese population (OR = 4.04, 95% CI: 1.77−9.25). Furthermore, the virulence factors of *H. pylori*, such as *CagA* and *VacA*, may not be involved in the mechanisms through which they promote the development of ACS. This finding provides additional evidence for the association between *H. pylori* and ACS across different ethnic groups while refuting the biological mechanism by which *H. pylori* affects ACS through lipid metabolism regulation. Regular screening for *H. pylori* and eradication treatment in elderly individuals and those at high risk for ACS, such as patients with hypertension, long-term smokers, diabetics, and individuals with hyperlipidemia, may be effective strategies for reducing the incidence of ACS. Individuals at low cardiovascular risk who are infected with *H. pylori* may not require strict lipid management. As a result, we recommend relaxing the indications and eligible populations for *H. pylori* eradication therapy, as this may offer valuable insights for the treatment guidelines of *H. pylori* for example, considering atherosclerosis as a new indication for eradication therapy could prove effective for long-term ACS prevention. Enhancing the promotion of the association between *H. pylori* and cardiovascular diseases, along with providing discounts for *H. pylori* testing and treatment for the elderly population, will effectively reduce the prevalence of *H. pylori* infection among high-risk groups for ACS. Although widespread eradication therapy for *H. pylori* may increase its potential resistance rates, the health benefits to high-risk populations for cardiovascular diseases may be more significant. Moreover, reducing the infection rate of *H. pylori* is a fundamental measure to decrease the risk of ACS caused by this bacterium. To this end, the development and promotion of *H. pylori* vaccines may be an effective strategy to reduce the number of infected individuals. Nevertheless, owing to the limited sample size, the inability to comprehensively control confounding factors, and the inherent limitations of observational studies, it is crucial to conduct multicenter randomized controlled trials in the future to validate the true association between *H. pylori* and ACS. Additionally, exploring host genetics and the effects of *H. pylori* on the gut microbiota represents an interesting direction for future research on the biological pathways associated with *H. pylori* and ACS.

## Data Availability

The raw data supporting the conclusions of this article will be made available by the authors, without undue reservation.
